# Blood DNA methylation and type 2 diabetes mellitus: A protocol for systematic review and meta-analysis

**DOI:** 10.1097/MD.0000000000020530

**Published:** 2020-06-05

**Authors:** Xian Wang, Jiao Yang, Xianliang Qiu, Qing Wen, Min Liu, Qiu Chen

**Affiliations:** a School of Clinical Medicine; b Department of Endocrinology, Hospital of Chengdu University of Traditional Chinese Medicine, Chengdu, China.

**Keywords:** Candidate-gene DNA methylation, epigenetics, Genome-wide DNA methylation, Global DNA methylation, Peripheral blood, type 2 diabetes

## Abstract

**Background:**

Epidemiology of type 2 diabetes (T2D) is affected by genetic and environmental factors. Deoxyribonucleic acid (DNA) methylation as a kind of epigenetic mechanism has been reported to affect the pathogenesis of T2D. Both global DNA methylation and candidate genes were observed differentially methylated and identified changes. Peripheral blood biomarkers could have significant clinical utility due to the noninvasive operation and showed efficacy in measuring T2D-related DNA methylation. Moreover, due to the reversible feature of DNA methylation, it can be an effective therapy for T2D.

**Methods:**

This study will be conducted according to the preferred reporting items for systematic review and meta-analysis protocols 2015 statement. The protocol of this systematic review and meta-analysis was registered on international platform of registered systematic review and meta-analysis protocols (No. 202040136). The following electronic bibliographic databases will be searched from inception: EMBASE, MEDLINE, Web of Science, Cochrane Central, China National Knowledge Infrastructure, Wanfang and China Science and Technology Journal Database, and Clinical Trials (ClinicalTrials.gov). A search strategy will be developed using a combination of medical subheadings words and keywords related to T2D, human peripheral blood, and DNA methylation. Two reviewers will screen the literature, and a third reviewer will adjudicate in case of disagreements. All the data will be extracted by 2 independent reviewers. The Cochrane Risk of Bias Assessment Tool will be used to assess the bias of randomized controlled studies, and the Newcastle–Ottawa scale for nonrandomized controlled studies. All the assessment will be investigated by 2 independent reviewers. The overall quality of extracted data will be assessed by using the grading of recommendations, assessment, development, and evaluation assessment tool. Disagreements and conflicts will be resolved by discussing it with a third reviewer. The narrative synthesis and meta-analysis of all evidence will be performed as the main findings analyzed.

## Abstract 2

Supplemental Digital Content is available in the text

## 1 Introduction

With the increase in obesity, energy-rich diet, physical inactivity, diabetes mellitus is leading to increased morbidity, disability, and death globally. In 2019, 463 million people were estimated to have diabetes, and the projection to 2045 is around 700 million.^[1]^ Type 2 diabetes (T2D) is the most common type of diabetes, accounting for around 90% of all diabetes over the world.^[1]^ The characteristics of T2D include increased hyperinsulinemia, insulin resistance, and pancreatic β-cell failure with up to 50% cell loss at diagnosis.^[2]^ Furthermore, T2D may develop micro- and macro-vascular complications with a long asymptomatic phase, which causes both mental and socioeconomic pressures.^[3]^

Epidemiology of T2D is affected by genetic and environmental factors. Genome-wide association studies (GWAS) have led to the identification of common variants of glycemic genetic traits for T2D; however, these only accounts for 10% of total trait variance.^[4]^ Epigenetics is defined as heritage changes in the genome without altering the genomic sequence, including deoxyribonucleic acid (DNA) methylation, chromatin modification, and noncoding ribonucleic acids.^[5]^ DNA methylation involves the covalent addition of a methyl group to carbon C5 of cytosine nucleotides to create 5-methylcytosine,^[6]^ and mostly associated with cysteine-phosphate-guanine sites located in the promoter regions.^[7]^ The process is catalyzed by the DNA methyltransferase enzymes, and S-adenosyl-methionine is the methyl donor.^[6]^ Ultimately, the transcription may be suppressed by modulating the binding of transcription factors to DNA, and through the recruitment of methyl binding proteins and transcriptional corepressors.^[7]^ Furthermore, DNA methylation is reversible so that drug targets can depend on DNA methylation modification.^[8]^

Previous studies have revealed the association between DNA methylation and T2D. Human tissues from T2D patients and non-T2D donors were examined to distinguish the differences of DNA methylation and expressions, such as pancreatic islets,^[9–12]^ skeletal muscle,^[13,14]^ liver,^[15]^ and adipose tissue.^[16]^ Dozens of relevant genes were observed differentially methylated and identified changes with impaired β-cell function, insulin secretion, impact on lipogenesis and adipokine secretion, insulin sensitivity. These studies revealed that the risk factors of T2D, such as obesity and aging, can affect the methylome in nondiabetic subjects, leading to the development of insulin resistance, impaired insulin secretion, and T2D.

Although the studies in DNA methylation of pancreatic β-cells and insulin-response tissues had been explored widely, the possibilities to monitor human tissues in vivo are limited in clinical practice. However, peripheral blood biomarkers could have significant clinical utility due to the noninvasive operation and blood collection is a routine medical checkup. Significantly, the changes in T2D-related DNA methylation in pancreatic β-cells and insulin-response tissues have been reported in the blood, revealing a number of genes involved in glucose and lipid metabolism, insulin secretion and function, pancreatic and cardiovascular function, and gut microbiota.^[17–23]^

Nevertheless, due to the different cell types (erythrocytes, basophils, neutrophils, eosinophils, monocytes, lymphocytes, etc), different methods for isolating DNA and measuring DNA methylation, the previous studies showed large heterogeneity and different DNA methylation trends. Meanwhile, the small sample size of previous cross-sectional or case-control studies would limit the power of test. As a result, it is necessary for carrying out a systematic review and meta-analysis to assess if blood DNA methylation can be a risk factor, possible biomarker or prognostic marker for T2D.

## 2 Objectives

This systematic review aims to assess and summarize the studies which examine the relationship between blood DNA methylation and T2D, including global DNA methylation, candidate-gene methylation, and GWAS. Indeed, this study will explore the possibility of blood DNA methylation as a biomarker and intervention targets for T2D.

## 3 Methods

The protocol of this systematic review and meta-analysis was submitted on international platform of registered systematic review and meta-analysis protocols (No. 202040136) which could be available on https://inplasy.com/. This study will be conducted according to the preferred reporting items for systematic review and meta-analysis protocols (PRISMA-P) 2015 statement.^[24]^

### 3.1 Eligibility criteria

This systematic review and meta-analysis will identify and summarize the studies which examine blood DNA methylation in T2D and controlled human subjects. The studies published in Chinese, English, and other languages that can be translated through Google Translate will be considered. In addition, the review will include global DNA methylation studies, candidate-gene methylation studies, as well as GWAS. Any randomized controlled, longitudinal, cross-sectional, and case-control studies with sufficient information will be included.

### 3.2 Study identification

The following electronic bibliographic databases will be searched from inception: EMBASE, MEDLINE, Web of Science, Cochrane Central, China National Knowledge Infrastructure, Wanfang and China Science and Technology Journal Database. Meanwhile, Clinical Trials (ClinicalTrials.gov) will also be searched. The included studies will also be hand-searched to identify other potentially eligible studies. In addition, it is essential for contacting the authors if there is incomplete or misunderstanding information.

### 3.3 Search strategy

A search strategy will be developed using a combination of medical subheadings words and keywords related to T2D, human peripheral blood, and DNA methylation. The medical subheadings words include “diabetes meillitus, type 2” and “DNA methylation.” The following keywords will be used: “human,” AND “blood,” OR “peripheral blood” OR “peripheral blood mononuclear cells” OR “peripheral blood leukocytes” OR “peripheral blood lymphocytes” OR “white blood cells.” A sample search strategy for PubMed is shown in Supplementary Table 1.

### 3.4 Study selection

**Figure 1:**
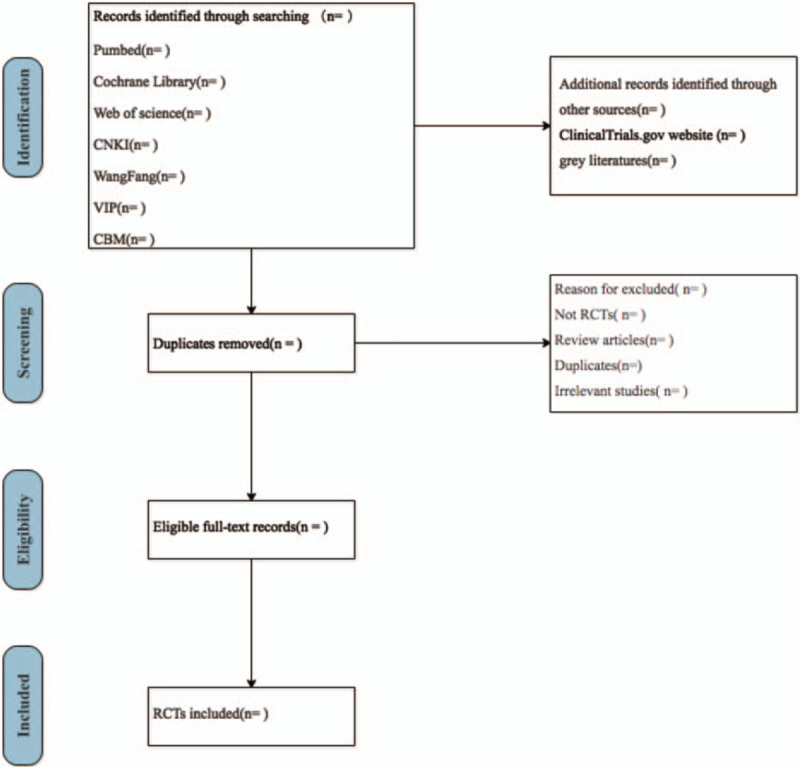
Flow diagram of study selection.

For eliminating the discrepancies and inconsistencies, a pilot between reviewers will be conducted to ensure adequate comprehension. Two reviewers (XW and JY) will screen the literature through identifying all titles, abstracts, and selected full-text for eligibility. In case of disagreements, the third reviewer (XLQ) will adjudicate. Reviewers will document the reasons for exclusion and the whole eligible process will be shown in the PRISMA flow diagram (Fig. 1).

### 3.5 Data extraction

The descriptive details will include authors, population, sample size (T2D group vs controls group), gender, biological source, DNA methylation methods, and outcomes in Global DNA methylation studies, investigated genes, and tissue types will be additionally documented in candidate gene methylation studies and GWAS studies. All the data will be extracted by 2 independent reviewers.

### 3.6 Assessment of study quality

The Cochrane Risk of Bias Assessment Tool will be used to assess the bias of randomized controlled studies,^[25]^ and the Newcastle–Ottawa scale for nonrandomized controlled studies.^[26]^ All the assessment will be investigated by 2 independent reviewers. The overall quality of extracted data will be assessed by using the grading of recommendations, assessment, development, and evaluation assessment tool.^[27]^ Disagreements and conflicts will be resolved by discussing it with a third reviewer. Funnel plots will be performed to assess the publication bias if more than 10 studies. The systematic review and meta-analysis will be reported in accordance with PRISMA guidelines.^[28]^

### 3.7 Quantitative synthesis

Review Manager V.5.3. will be used to analyze the statistical data. The *I*^2^ statistic and *X*^2^ test will be measured to analyze the heterogeneity, whose cut-off value is 50% and 0.10, respectively. A fixed-effect model will be used for the meta-analysis with moderate heterogeneity (*I*^2^ < 50%), otherwise a random-effects model will be performed to account for between-study heterogeneity. Odds ratio and 95% confidence intervals will be calculated to assess the association between blood DNA methylation and T2D. Rate ratios or hazard risks will be extracted in cohort studies.

## 4 Discussion

Previous studies showed the correlation between blood DNA methylation and T2D. However, limited evidence was demonstrated for the diagnostic and therapeutic potential of blood DNA methylation. This systematic review aims to summarize and re-assess the data of past studies and explore the epigenetic mechanism of T2D. The findings may instruct future researches into the optimization of T2D's management programs using epigenetic markers as part of a screening tool.

## Author contributions

XW, JY conceived the idea and XW, JY, XLQ designed the study. QW and ML reviewed scoping searches and contributed to the methodological development of the protocol. XW, JY drafted the initial manuscript and all the authors (XLQ, QW, ML, QC) revised the manuscript. All the authors have given approval of publishing. QC is the review guarantor.

## Supplementary Material

Supplemental Digital Content
