# Supplementary material for: Blood DNA methylation and type 2 diabetes mellitus: A protocol for systematic review and meta-analysis
Source: Medicine (Baltimore). 2020 Jun 5;99(23):e20530. doi: 10.1097/MD.0000000000020530 (PMC7306281; doi:10.1097/MD.0000000000020530)
Supplement: Supplemental Digital Content [file medi-99-e20530-s001.doc]

**Table 1.PubMed search strategy**

| **Table1**  **Example** **of** **PubMed search strategy** | |
| --- | --- |
| **Number** | **Search terms** |
| #13  #12  #11  #10  #9  #8 | (#11) AND (#12)  (human) OR (humans)  (#3) AND (#10)  (#8) AND (#9)  ((((((blood cells[MeSH Terms]) OR (blood[Title/Abstract])) OR (peripheral blood[Title/Abstract])) OR (peripheral blood mononuclear cells[Title/Abstract])) OR (peripheral blood leukocytes[Title/Abstract])) OR (peripheral blood lymphocytes[Title/Abstract])) OR (white blood cells[Title/Abstract])  (#4) OR (#7) |
| #7 | (#5) AND (#6) |
| #6 | (DNA[Title/Abstract]) OR (Deoxyribonucleic acid[Title/Abstract]) |
| #5 | (((methylation[Title/Abstract]) OR (methylations[Title/Abstract])) OR (hypomethylation[Title/Abstract])) OR (hypermenthylation[Title/Abstract]) |
| #4 | DNA methylation[MeSH Terms] |
| #3 | (#1) OR (#2) |
| #2 | ((diabetes[Title/Abstract]) AND (mellitus[Title/Abstract])) AND (type 2[Title/Abstract]) |
| #1 | type 2 diabetes mellitus[MeSH Terms] |
